# Distance Learning in Pandemic Age: Lessons from a (No Longer) Emergency

**DOI:** 10.3390/ijerph192316302

**Published:** 2022-12-05

**Authors:** Loredana Addimando

**Affiliations:** Department of Teaching and Learning, University of Applied Science and Arts of Southern Switzerland, 6600 Locarno, Switzerland; loredana.addimando@supsi.ch

**Keywords:** teaching in pandemic age, emotion in teaching, distance learning

## Abstract

For the first time in the history of the global school system, the adoption of distance education modalities became necessary in response to the measures and restrictions implemented to stem the global pandemic generated by COVID-19. Online learning is not a new topic in education; it refers to studying for a certificate using online platforms that offer online courses rather than visiting lectures. Distance learning is a type of training that involves online learning under the supervision of a classroom teacher, but it can still be a little-known modality for both teachers and students. Preparing students for interactions and emotion management is essential in any teaching mode to maximize the learning and participation of the entire class group. This issue becomes even more critical in distance learning because it lacks those aspects of immediacy and concreteness typical of face-to-face interaction. The present article attempts to review the impact of distance learning versus traditional education brought about by the forced experience of distance learning due to the pandemic. In summary, this research has provided some initial findings about distant learning research during the pandemic age. To have a successful learning experience, students must be aware of their responsibilities and master their areas of autonomy, emotions, and feelings. Teaching at a distance is a challenge. As a general rule, learning activities that are successful in a traditional classroom may be adapted to the distance learning setting, but this will take more than a few minor tweaks to the slides or handouts. In order to engage pupils and maintain their attention and motivation levels, these techniques will probably call for more imaginative and inventive ways.

## 1. Introduction

In relation to worldwide pandemics and epidemics, one of the most historically significant societal transformations has been caused by the severe acute respiratory syndrome (SARS) linked coronavirus (SARS-CoV-2) known as COVID-19. People’s behavior is changing quickly and obtrusively in various societies [1]. Due to the pandemic, many countries had to restrict some of their inhabitants’ personal freedoms in the first two months of 2020. In this instance, using technology for work, social interaction, and leisure momentarily took the role of routine everyday tasks. This quick transition affected society as a whole with a profoundly psychological effect, affecting people’s mental health [2], how firms conduct themselves and, consequently, the global economy [3]. In people’s professional lives a severe disruption has occurred, and there is a greater reliance on technologies to keep industries and system of commerce alive. This review is moving through the literature on effects of this rapid adoption of technology with a focus on the relation between emotion and learning, specifically on distance education in compulsory school education sector.

For the first time in the history of the global school system, the adoption of distance education modalities became necessary in response to the measures and restrictions implemented to stem the global pandemic generated by COVID-19. Despite a decrease in severity, it is significant to highlight that the WHO has not yet declared the pandemic to be over. Because of this situation, school site directors and teachers were forced to operate under entirely unforeseen conditions, accommodating organizational plans aligned to the different risk scenarios of contagion spread that gradually unfolded over time.

Understandably, teachers at school experienced mixed emotions and feelings, alternating between skepticism, fear, and enthusiasm. The pandemic crisis has somehow forced schools and training institutions to accelerate the process of digitizing the infrastructure and practices of school teaching at all levels, requiring everyone to make a great organizational and conceptual effort in a short time.

Between the confusion of some and the optimism of others, we find the responsible and mature attitude of many, who, in their role as principal, teacher, or parent, have not underestimated the need for precautionary measures to contain the contagion but have nevertheless tried to safeguard as far as possible a “school at school”, attempting to make the education of students sustainable even in a context profoundly modified by emergency constraints.

In this context, we have seen the emergence of the potential of hybrid forms of teaching (in the presence and at a distance) that have allowed a new range of possible educational actions and, at the same time, have protected the importance of direct contact between teachers and students, safeguarding in many cases the emotional aspects that support motivation and consequently learning.

Beyond external, unexpected, and/or emergency needs, not dependent on the teacher’s will, the moment of necessity that has led to the increase in the use of digital tools in teaching has made it possible to experience an aspect of contemporary didactics that finds a flexible resource in technologies. Along with traditional classrooms, online learning systems like Khan Academy, Net Ease Cloud Class, and Massive Open Online Courses (MOOC) are also available to students. Teachers are becoming more conscious of the crucial role that online education and learning plays for students who are unable to attend in-person schooling, especially those afflicted by the Corona Virus Disease 2019 (COVID-19) [4]. However, educational institutions are once again using classroom instruction rather than simply remote learning, but these methods have nonetheless become a part of the teachers’ toolkit of activities. Online learning gives students access to learning opportunities wherever they are, and it also makes the learning processes more student-centered [5].

Many teachers revealed that the most critical work was redesigning the educational interventions and remodeling the teaching units globally. In addition to the cognitive aspects of technology adoption, such as those examined by the unified theory of acceptance and use of technology [6], which moved from the technology acceptance model [7] and from the Affordance Theory [8], researchers contend that emotions can also have an impact on how technology is used and adopted [9,10,11]. For instance, studies have shown that the desire to utilize new technologies can be negatively impacted by fear and anxiety, but favorably by feelings of excitement, arousal, and pleasure, e.g., [12,13,14].

Treasuring the results achieved will be possible to the extent that we will review the experiences made in a collective dimension of awareness of opportunities and risks that can be taken into account and reworked by the entire “educating community” [15]. Teachers’ experience and professionalism are indispensable because, with or without technology, schoolwork can produce solid and lasting learning, even in an emergency.

As a result, we are observing an increase in the number of teacher communities that use technology to facilitate networking and asynchronous working [16]. School teachers are increasingly creating learning environments through the use of internet forums and platforms, as well as haphazardly creating virtual areas where they may deposit and trade content [17,18], but there is still a lack of an organic vision for school policies that can encourage the development and spread of these communities of teachers and students [19].

The literature agrees that many didactic actions acted in a technological environment may prove, when rigorously designed, more helpful to learning than when proposed in the presence [20], as well as some teaching practices that may not find a counterpart online and, when adapting to the new tool, in some cases, lose effectiveness [21]. In any case, the invitation that comes from many bodies [22,23] is not to oppose in-presence teaching and distance teaching, but on the contrary, to work so that in the future, increasingly there is not one without the other (as well as removing the difference between teaching through technologies and traditional teaching).

### Aim and Methodology

The article attempts to review the impact of distance learning versus traditional education brought about by the forced experience of distance learning due to the pandemic.

Although the pandemic has regressed in severity, its end has not yet been officially declared. It is also evident that the previously implemented restrictive measures are currently being reduced. As a result, it seems reasonable to attempt to list some evidence that we have already started to recognize.

Without claiming to be exhaustive, the study aims to highlight a few instances of actions taken by educational institutions to strengthen and advance the current distance learning technologies. By considering the effectiveness of distance education on the one hand, and the trajectories that can be glimpsed to consolidate the efforts made so far on the other, the goal is to give the reader a reasonable perspective on how to turn the limitations imposed by the pandemic into new opportunities for the development of teaching/learning processes. The development of the contribution is done in accordance with the three fundamental axes of reasoning.

In light of new and post-new demands, there is a need to redefine distance education’s bounds. The article is evidence-based and addresses the three fundamental axes of reasoning.

- An evidence-based discussion on the need to redefine distance education’s limitations in light of new and post-new demands, addressing the following question: What does distant learning entail following COVID-19?
- A concise analysis of the efficiency of distant education
- An analysis of post-pandemic action cases that we may now see as valuable for implementing remote learning, like the value of professional learning communities.

The literature review was guided by the following inclusion criteria: (1) studies into online and in-person hybrid classrooms during the pandemic; (2) investigations into psychological issues like the emotional and affective states triggered by the use of distance learning technologies during the emergency.

## 2. What Is Distance Education after the COVID-19? A Brief Literature Review

Distance education has become an essential topic of study in education and training over the past two decades. Nowadays, most education conferences deal with some aspect that has to do with e-learning, and there is a lot of research that has been questioned over the years about the effectiveness of educational technologies in the learning/teaching process. Indeed, the world of distance education is promising, and recent hardware and software innovations are making distance education systems more available, easier to use, and less expensive. Let us think about the new possibilities applied by artificial intelligence to teaching, by reality augmentation for creating new learning environments, up to gaming and educational robotics. Training and distance education, also encouraged by ongoing technological development, have entered the mainstream of education nationally and internationally. Numerous online courses of self-training are made available by prestigious universities worldwide, and even more numerous are the software applications to manage even huge audiences of learners (think of MOOCs). In order to be truly immersive, these devices must take into account not only the cognitive aspects but also the emotional experience offered to the user. From an organizational perspective, research has demonstrated that when distance learning is introduced in an organization, employees can experience strong emotions that determine their adoption behaviors. For example, ref. [24] studied the relationships between employee emotions and workplace distance learning technology using components of the Regulatory Focus Theory and Affective Events Theory. The authors argued that employees’ emotions surrounding the deployment of the new technology can affect their responses to technology adoption (e.g., resistance to change or rejecting the new system). More recently, Mamun and colleagues [25] employed the Expectation–Confirmation Model to investigate Information Technology (IT) use in the workplace, explaining that emotions are of paramount importance for the employee’s continued use of technologies for learning.

Beyond the ongoing opposition in both business and education, the current issue is no longer whether or not to do distance learning, but how to do it better.

However, what does distance learning mean in the current time? Furthermore, how do users’ emotions in the post-pandemic age influence it?

These still appear to be far from marginal questions. In the first place, distance has multiple meanings. The distance can mean geographical distance, temporal distance, and perhaps intellectual and emotional distance.

Whenever we talk about distance education, we should first understand what kind of distance we mean. Furthermore, different “distances” can be simultaneous or alternative. We can be present physically and distant intellectually, we can be in the here and now physically and intellectually, or we can be very closely interacting all the time, though separated in time and space.

Second, the term “distance education” has been applied to an enormous variety of educational programs and actions that reach large audiences through a wide variety of media. First, there were paper-based materials to train and educate at a distance, and then telecommunications. Finally, technological changes have pervaded the traditional ways in which distance education was conceptually defined.

Coldeway [26] provided a valuable framework to help redefine, within the space–time dimension, four ways in which education can take place.

Traditional education typically takes place at the same time and in the same place (ST-SP). This approach to teaching refers back to the typical self-contained classroom, which is most often teacher-centered. ST-SP teaching involves teachers and learners being present at the same time in one physical location.

Different times, same place (DT-SP) means that individual learning takes place within school walls and that the school organizes multiple sections of the same classes so that students can choose the location and time of classes to attend. Additionally falling under these approaches are those instructional actions available at different times of use to students but implemented in the same place, examples of which are learning environments such as the library or computer lab. The last two categories focus, instead, on teaching that takes place in different places. Teaching can be delivered in different locations simultaneously (ST-DP) when teachers and students use, for example, telecommunications systems. Often, video conferencing connects the classroom to the teacher and students to students at a distance. Increasingly, video conferencing systems (e.g., Zoom, Microsoft Teams, Google Meet, etc.) are being used to provide live teaching. This approach is also called synchronous distance learning. Finally, with the help of, e.g., audio and video media (think of podcasts and webinars), teaching can take the form of different times and places (DT-DP). Students can learn even if they are at different times and in different places. Coldeway [26] argues that the purest form of distance education occurs at DT-DP. In other words, students choose when and where to learn and when and where to access learning materials. This approach is also called asynchronous distance learning. Zoom, Blackboard Collaborate, Elluminate, Adobe Connect, and Webex are some of the synchronous online technologies prevalent in higher education [27]. They render immediate feedback and allow for multi-modality communication [28].

In 1981, Weinstein [29] carried out research on the suitability of the instructor’s chosen teaching method with the environment in which instruction is conducted. Later, others stressed the importance of taking into account the close connection between subject, body, and environment in learning processes [30,31], which is even more important in virtual environments because we do not have access to all the physical elements present in physical environments and frequently have to use vicarious solutions to make up for their absence [32]. In online learning environments, it is crucial to encourage and maintain a variety of interactions amongst users, since these features of interaction and emotion are crucial for determining the success and quality of online education [30].

## 3. Results on the Effectiveness of Distance Education in Pandemic Age

School is a complex system that is able to manage complex problems. Complex problems have the unfortunate property of being immaterial and fading away as soon as one attempts to subject them to basic analytical processes.

Nevertheless, this complexity is rarely observed from a systems perspective (and even less frequently from an emotional perspective). More often, the impetus for action comes from specific contingencies (such as a pandemic) or biased readings of contexts.

Systemic theory helps to observe phenomena within a frame of reference that considers both the local and the global. Among the first to talk about complex systems, Von Bertalanffy [33] stated “a system can be defined as a complex of interacting elements. Interacting means that the elements (P) are connected by relations (R) so that the behavior of Pin R is different from what would be its behavior concerning another relation R” (p. 97). The definition offers at least two pieces of information: a system is composed of several elements, and an element can be in turn a system, like in the case of living systems (cells are systems, composed of elements called molecules, but also that molecules are systems composed of elements called atoms, etc.). Thus, a hierarchical fit can exist between systems.

Similarly, technologies should be included in the education and training system, integrating technical and content aspects in a hierarchical and synergistic manner with respect to other typical educational instances.

A recent study [34] explores the psychological impact of technology adoption during the first year of the COVID-19 pandemic on UK Higher Education (HE) employees. Using sentiment analysis, authors analyzed approximately 9000 tweets focusing on technology use in UK HE between March 2020 and February 2021, leading to the identification of significant changes in perceptions and feelings. The transition from emergency/rapid to planned/proactive technology adoption and integration is associated with the emergence of a number of distinct positive (e.g., empowerment and self-efficacy) and negative (e.g., isolation and stress) psychological consequences in each phase of the pandemic, according to the authors. Such studies provide a framework for illustrating how employee emotions are impacted by technology adoption and how these feelings alter as a result of the shift from rapid to planned technology adoption. The ramifications of investigations on emotion in distance learning therefore provide invaluable insights for schools transitioning from emergency to planned technology adoption in the future, given the significance of emotions inside organizations, particularly in groups who teach.

### 3.1. Empirical Evidences of Distance Education Effectiveness

Whether distance education is comparable to a traditional form of education has long been a debated topic, and thanks in part to the accessibility of large amounts of previously inaccessible data, research has made strides in studying the interaction between instructional, educational, and hybrid forms of in-person and distance education technologies [35,36].

Beyond the empirical research evidence, however, two themes must be addressed that seem to support a clear preference for traditional teaching, even in the variety of its different forms. When teachers talk to students, the latter say that distance learning would not be their first choice [37]. They prefer to meet in groups, delve deeper with the teacher in the classroom, and do the hands-on activities in the lab; they report that they value the presence of a learning team and that the informal interactions are valuable components of the learning experience as a whole [38]. Nevertheless, some surveys show that even very young students are increasingly asking for distance learning. They want to be able to supplement and even replace conventional learning experiences with distance education experiences. This experience allows them to gain autonomy and motivation to learn where and when [39]. The learning space, in this sense, expands and encompasses essential dimensions that have to do with the uses and effectiveness of e-learning and distance education in general. Students’ learning engagement is today recognized as a critical indicator in the evaluation of distance learning [40].

In a thorough review of the literature, Simonson et al. [41] concluded that “distance education is not different education, it is distance education” (p. 124), and that “research clearly shows that distance education is an effective method for teaching and learning” (p. 139). Another impressive body of work which has attempted to systematize research on the topic is Moore and Diehl’s Handbook of Distance Education [40] which is now in its fourth edition. Among the many pieces of evidence highlighted in this handbook, perhaps one of the most critical passages is the one in which, thanks to an extensive review of recent studies, it is possible to conclude that the use of distance and technologies, in general, does not have a significant impact on learning per se. From this point of view, some authors, and not without polemic verve towards the enthusiastic supporters of educational technologies, call for caution in being too blindly attracted to everything technological. For example, Clark [42,43] provocatively said in an article in the leading scientific journal Review of Educational Research [42] that: “The best evidence today is that media are mere vehicles that deliver instruction, but they do not affect student achievement any more than the truck that delivers groceries does”, (p. 445) and then years later, picking up on the concept in his text titled Learning from media: Arguments, analysis, and evidence [43] stated that: “It is likely that when instructional actions are undertaken using different media produce similar learning outcomes, the cause may be sought in the method that the two treatments have in common…abandon your enthusiasm for the belief that media attributes cause learning” (p. 28).

During the growth and diffusion of the distance education content in the United States, the Association for Educational Communications and Technology concluded that comparative research studies of learner academic achievement tended to show no significant differences between different delivery systems and between distance and traditional education [18]. On the contrary, for higher schools and tertiary education, much of the data were promising in indicating significantly higher achievement in those who learned at a distance. In other words, it is not whether an instruction is delivered in a traditional, face-to-face, or distance setting that predicts learning [43,44]. Consequently, training teachers in effective instructional strategies is critical for a school that makes a “virtue of necessity” and permanently integrates modes of working at a distance with learners due to unresolved emergent health care frameworks.

In order to do this, there must be maximum dissemination about the essential requirements, or hygiene factors [45], accompanying the development of distance learning modalities: distance-learning courses should be carefully designed for specific objectives and developed before the start of the course. It is important to use ways to visualize ideas and concepts when designing the delivery to be provided to distance learners. Schools must prepare appropriate support systems to provide distance students with access to resources and services. Interaction between faculty and students and among students must be facilitated and encouraged frequently. Assessment must be designed for the specific learning outcomes of the individual educational experiences constructed.

In the changing and diverse environment in which distance education is practiced, many questions remain unanswered. It is not easy to arrive at a definition or agree with a single theory on teaching and research on this topic in this environment. Technologies, globalization, and new ideas about student learning challenge established approaches to distance education practices. This theme of change is evident in discussions about distance education and its definition, history, status, and theories that can account for all the elements involved in new forms of teaching/learning.

In conclusion, the key insight we can draw from this brief analysis is that people should better comprehend the advantages of distance learning rather than dismiss it as a minor-league education, especially in light of the post-pandemic realities. Distance education can be as effective as any other type of education. Studies say that learning happens and knowledge is retained. The keys to the success of distance education are in the design, development, and delivery of educational content and are not related to geography or time. The implication of the arguments so far is that when new technologies emerge, they often enable users to be more efficient. However, it is not the technologies themselves that cause the changes; instead, the technological changes occur because of new ways of doing things enabled by the technologies.

### 3.2. Toward Shared Teaching: The Importance of Professional Learning Communities

A survey of students enrolled in online distance education programs for social work [46] demonstrated how emotions impact distance learning; the survey revealed that some students found it difficult to stay motivated and felt lonely. Therefore, constructivist pedagogy was tested, which included peer- and self-assessment of practice role plays. Data for students revealed an increase in the frequency with which they used interactive technology and accessed online readings. Observing that only the cohort of distance learners spontaneously communicated the content of what they had actually learned, despite the two cohorts of students participating expressing favorable experiences with their learning. These results imply that the continual peer contact brought about by the new approach led to a more profound and long-lasting learning experience.

As a result, teachers should be able to develop learning environments using online forums and platforms. Although many countries throughout the world have been digitizing their educational systems in recent years, these kinds of changes in the ways that teachers educate in the classroom require support and training. Institutions can think of learning communities as a place to experiment with cutting-edge teaching methods and activities since they offer students the chance to participate in a virtual community.

Virtual communities for learning are ideal places in which to develop practices and share knowledge. They are described as a way of functioning in educational institutions that aims to create a comprehensive institute culture, and that encourages faculty to undertake and develop collective reflections aimed at the continuous improvement of their knowledge, skills, and teaching practices in order to respond to the diverse needs of learners and to make learners achieve better educational outcomes [47,48,49].

Within this framework, Bonaiuti [50] distinguished several possible aggregates:

- Dialogue communities (or virtual communities) are formed by a group of people who share practices, activities, and interests for the pleasure of doing so and who find the environment to meet, share experiences, and knowledge on the Internet. People who participate in a virtual community benefit from exchanging information on a given topic and benefit from the system of relationships established while attending the community.
- Communities of practice [51] are based on the idea that learning consists of negotiating new meanings in a participatory interaction. In this sense, learning is an essentially experiential and social process, creates emergent relational structures, and contributes to the construction of professional identity through community membership.
- Learning communities [52]: they are based on the solid sharing of knowledge by the participants and the enhancement of metacognitive aspects (learning to learn) of the learning process. Among the privileged learning models, there is, in fact, the cognitive apprenticeship, which is based on the idea that the learner observes the master and imitates them (modeling). The master assists and facilitates the work (coaching), provides support in terms of stimuli and resources (scaffolding), and finally progressively decreases the support provided to leave more autonomy to the learner (fading).

With the focus shifting from a technological to a social aspect, the research of distance learning has evolved into the study of online learning interaction [53,54,55]. Creating an online learning community involves both faculty and students [56,57]. According to Bernard et al. [58], a community will be strong if its members help to shape its surroundings. Members should establish rules and regulations and clearly define the community’s purpose. This community will remain intact until everyone can see how important it is for them to help one another. Second, we require capable teachers. In addition to managing the community, teachers must also take on the role of facilitators. The third rule is that it is encouraged to tell personal stories. Personal narrative, according to Bakker and colleagues [59], is the light that helps societies thrive. Everyone should take an active role in developing a collegial learning situation. Students and teachers must understand their role in learning experiences [60,61]. Respect for others is an integral part of group work, especially distance [40]. In an online interaction, students may need instruction on communication protocols (or netiquette). Students need to be prepared to use microphones or other equipment in a learning environment using an audio resource (a podcast, for example). In addition, they need to understand their responsibilities to be courteous and well-behaved in communications, both with the teacher and their peers. Similar to what happens in a real-world setting, and even more so where distance poses constraints, in an online environment, students need to be sensitive to their peers and carefully choose appropriate language to express themselves. Moreover, they may need to be informed of any issues, cultural, social, and current affairs that might be important at a given time, thinking of the historical moment we are living and the need we will have to reframe it (also) in school in the coming months and years.

Finally, developing hybrid activities and practices can be successfully accomplished through the usage of learning communities. The hybrid approach focuses on creating a cohesive learning experience which combines face-to-face sessions with online learning materials and activities. Before being employed and spread permanently, these activities need to be carefully planned and require space for testing. A learning community could be a safe environment where they might experiment with teaching with other faculty members or students. For instance, according to some authors [62,63], it is possible for students to engage in “pedagogy of discomfort” in learning communities. The pedagogy of discomfort is a process of self-examination that requires students to critically engage with their ideological traditions and ways of thinking about issues such as racism, oppression, and social injustice. This approach fosters self-reflection in students by addressing affective, cognitive, and emotional needs. Emotions are powerful tools for learning also because learning activities purposefully designed to challenge preconceived assumptions can develop students’ understanding of their place in power dynamics within society. During this reflective process, students (and teachers) may experience a variety of emotions, including uncomfortable ones. This process can be a catalyst for change because the emotions it evokes might challenge conventional ways of comprehending a subject and assumptions made about it. Advocates of this pedagogy believe that emotions should be part of learning, and that if students are emotionally invested, transformation is more likely to occur. Learning communities offer a setting where educational initiatives to bridge social gaps or improve abilities that can have an impact on students’ wellbeing and mental health can be implemented. By educating students on how to take care of their own mental health, identify potential illnesses, and understanding how, where, and when to seek help, wellbeing education aids in the development of mental health “literate” in students. By referring to systems that can assist students as they navigate the educational system, wellbeing education goes beyond students and the development of pertinent knowledge, skills, and competences. A student-centered environment that encourages wellbeing and overcomes barriers to wellbeing in areas like the cultural realities of learners is one of these. As a result, the learning process incorporates qualities like compassion and empathy, promoting both the wellbeing of teachers and their students.

## 4. Discussion

Distance learning can still be a little-known modality for both teachers and students. Preparing students for interactions and emotion management is essential in any teaching mode to maximize the learning and participation of the entire class group. This issue becomes even more critical in distance learning because it lacks those aspects of immediacy and concreteness typical of face-to-face interaction. Students need to understand their responsibility and master their spaces of autonomy, emotions, and feelings to have a successful learning experience. Teaching at a distance is a challenge. The teacher must be creative and imaginative in course design and structure, seeking to place teaching practices and sustainability of educational pathways in a perspective of systemic complexity. A rule of thumb is that learning experiences that work in a traditional classroom may be adaptable to the distance-learning environment but will require more than a few minor changes to slides or handouts. These strategies will likely require more creative and innovative approaches to engage students and keep their attention and motivation high. For example, interesting innovations are present in the field of immersive technology and virtual reality [64,65]. Consequently, teaching at a distance can be an enjoyable experience for everyone involved. Keeping students’ interests high and motivating them to stay active can make teaching in distance education a worthwhile and enjoyable learning experience.

Attention to students’ needs, including emotional needs, is at the heart of a successful distance learning experience. Quality learning and practical learning experiences depend not only on the efforts and on the expertise of the teacher but are also primarily determined by the efforts and preparation of the students themselves. Teaching and learning are two sides of the same coin, and the learning experiences offered to students should be equivalent but not necessarily equal.

As many argue [40], the shift from the theoretical and practical dominance of traditional approaches to collaborative, creative, and innovative approaches supported by distance learning has created new challenges that have to do with maintaining a clear identity and coherence of purposes and goals of instructional actions.

At the end of the last century, there was an evident shift in thinking from the structural challenges of distance education to considering distance education as a possible method of teaching. The focus shifted from the structural constraints implicit in distance education to an emphasis on the educational experience. Recent developments blending online and face-to-face approaches have further blurred the distinction between the educational experience at a distance and the experience that occurs within school walls, thus contributing to hybrid forms that are difficult to categorize. Attempts to integrate online learning into the traditional paradigm include initiatives such as Massive Open Online Courses (MOOCs), augmented reality learning environments, or educational robotics. However, these initiatives still do not embrace all the possibilities of online collaboration. Traditional teaching is no longer a one-size-fits-all reality or constraint to adhere to but is transformed into the possibility of placing new tools and practices alongside established ones. This appears to be a classic example of a paradigm shift, as originally understood by Kuhn [66]. Meeting these challenges will involve a leadership function on the part of school administration through change toward innovation. Progress is only innovation until it becomes the new normal; leadership is needed until the new normal is achieved. This new normal will necessarily involve collaborative partnerships, networked environments, new models of teaching and learning, and ongoing strategic planning. One of the most significant challenges facing institutions is integrating technology into education at every grade level, whether traditional or distance learning is. We have an opportunity to put them at the center of the discussion, not only as an educational method, but as an engine for innovation and change. In this sense, learning communities can play an essential role in this process and can be valuable places of exchange and sharing, benefiting the entire educational community.

## 5. Conclusions

The purpose of the article is to illustrate how online education may be just as successful as traditional classroom instruction. The acceleration necessitated by the pandemic crisis has re-ignited the debate among academics and the general public. However, for the first time in educational history, the acceleration brought on by the pandemic has opened up previously unexplored spaces for work, as teachers’ and students’ evaluations of distance learning seem to emphasize. The emergency situation from which we have not yet emerged does not allow for the possibility of conducting studies on established distance learning educational actions. However, it is also clear that the overall severity of the previously put in place restrictive measures is currently decreasing. Therefore, it seems sensible to try and summarize some of the evidence that we have already begun to take notice of.

Currently, it appears that we are transitioning from a pandemic to an endemic phase, when COVID19 will be one of the most contagious diseases of our time. This suggests that we will need to change our lifestyles to comply with the health safety standards established by the epidemic (for example, the WHO, regardless of infection rates, continues to recommend certain good habits such as social distancing and systematic disinfection of people and workplaces). From the standpoint of distance education, this suggests that we might spend more time in the years to come implementing distance learning technologies as a fundamental and stable component of educational paths in every school, up to and including universities.

## 6. Limitations

This work is subject to several limitations. For instance, a meta-analysis could be used to more properly evaluate the efficacy of distance learning than a basic literature review. Furthermore, we are still in the emerging stage, so drawing firm conclusions may be inaccurate. Examining the research literature that has attempted to test the comparability between the two teaching methods during and after the pandemic, a number of methodological biases may be included with this work. For example, the non-comparability of the groups considered in the research analyzed, the impossibility of testing the effects of any intervention variables a posteriori, the heterogeneity of the educational situations taken into account, and so forth. This review attempts, while acknowledging these limitations, to critically reflect on the various viewpoints present in the most current scientific literature.

## Data Availability

Not applicable.
